# Fibrous dysplasia/McCune-Albright syndrome: state-of-the-art advances, pathogenesis, and basic/translational research

**DOI:** 10.1186/s13023-025-03909-8

**Published:** 2025-08-08

**Authors:** Biagio Palmisano, Camryn Berry, Alison Boyce, Julia F. Charles, Michael T. Collins, Alessandro Corsi, Fernando A. Fierro, Anne-Marie Heegaard, Hanne van der Heijden, Charles S. Hoffman, Chelsea Hopkins, Jaymin Upadhyay, Paul M. Wehn, Kelly L. Wentworth, Yingzi Yang, Xuefeng Zhao, Edward C. Hsiao, Mara Riminucci

**Affiliations:** 1 Department of Molecular Medicine, Sapienza University of Rome, Viale Regina Elena 324, Rome, 00161 Italy; 2 Department of Anesthesiology, Critical Care and Pain Medicine, Boston Children’s Hospital, Harvard Medical School, Boston, MA USA; 3 Metabolic Bone Disorders Unit, National Institute of Dental and Craniofacial Research, National Institutes of Health, Bethesda, MD USA; 4 Departments of Orthopaedic Surgery and Medicine, Brigham and Women’s Hospital, Harvard Medical School, Boston, MA 02115 USA; 5 Institute for Regenerative Cures, University of California Davis, Sacramento, CA 95817 USA; 6 Department of Cell Biology and Human Anatomy, University of California Davis, Sacramento, CA 95817 USA; 7 Department of Drug Design and Pharmacology, University of Copenhagen, Copenhagen, 2100 Denmark; 8 Biology Department, Boston College, 140 Commonwealth Ave, Chestnut Hill, MA 02467 USA; 9 G Protein Therapeutics, 3160 Porter Dr. Suite 250, Palo Alto, CA 94304 USA; 10 Division of Endocrinology and Metabolism, Department of Medicine, University of California, San Francisco. 513 Parnassus Ave., HSE 901, San Francisco, CA 94143-0794 USA; 11 Division of Endocrinology and Metabolism, Department of Medicine, San Francisco Veterans Affairs Health Sciences Center, San Francisco, CA 94121 USA; 12 Department of Developmental Biology, Harvard School of Dental Medicine, Harvard Stem Cell Institute, 188 Longwood Ave, Boston, MA 02115 USA; 13 State Key Laboratory of Oral Diseases, West China Hospital of Stomatology, National Clinical Research Center for Oral Diseases, Sichuan University, Chengdu, 610041 China; 14 Division of Endocrinology and Metabolism, Department of Medicine, The Institute for Human Genetics; and the Eli and Edythe Broad Institute for Regeneration Medicine, University of California, San Francisco, CA 94143 USA; 15 Oral and Craniofacial Sciences Graduate Program, School of Dentistry, University of California, San Francisco, CA 94143 USA

**Keywords:** GNAS, Rare disease, Skeletal stem cell, Mouse models, Bone remodeling, Osteoclastogenesis, Osteogenesis, RANKL

## Abstract

Fibrous dysplasia/McCune Albright syndrome (FD/MAS) is a rare genetic disease caused by postzygotic activating variants in the *GNAS* gene, encoding the α subunit of stimulatory G protein (Gα_s_). Although multiple organs may be involved, skeletal lesions usually represent the most severe and least treatable expression of the disease, leading to bone deformities, spontaneous fractures, and chronic pain that severely reduce patients’ quality of life.

The recognition of the causative Gα_s_ variants and the consequent ligand-independent activation of the adenylyl cyclase/cAMP/PKA pathway has provided a clear molecular explanation to most extra-skeletal pathologies of FD/MAS, leading to the development of effective therapeutic approaches. In contrast, a detailed understanding of the cellular and molecular mechanisms that act downstream of the Gα_s_ pathway to generate FD bone lesions and clinical expression thereof remain elusive. Multiple key issues remain to be addressed, including some questions that have recently emerged such as the interaction between mutated and non-mutated cells and the role of the latter in the development of the fibrotic tissue.

In this review, we provide a summary of the proof-of-concept, preclinical data, and experimental tools that have emerged to date from basic and translational studies on FD and represent the background for future research on the pathogenesis and treatment of this rare disease.

## Background

**Fig. 1:**
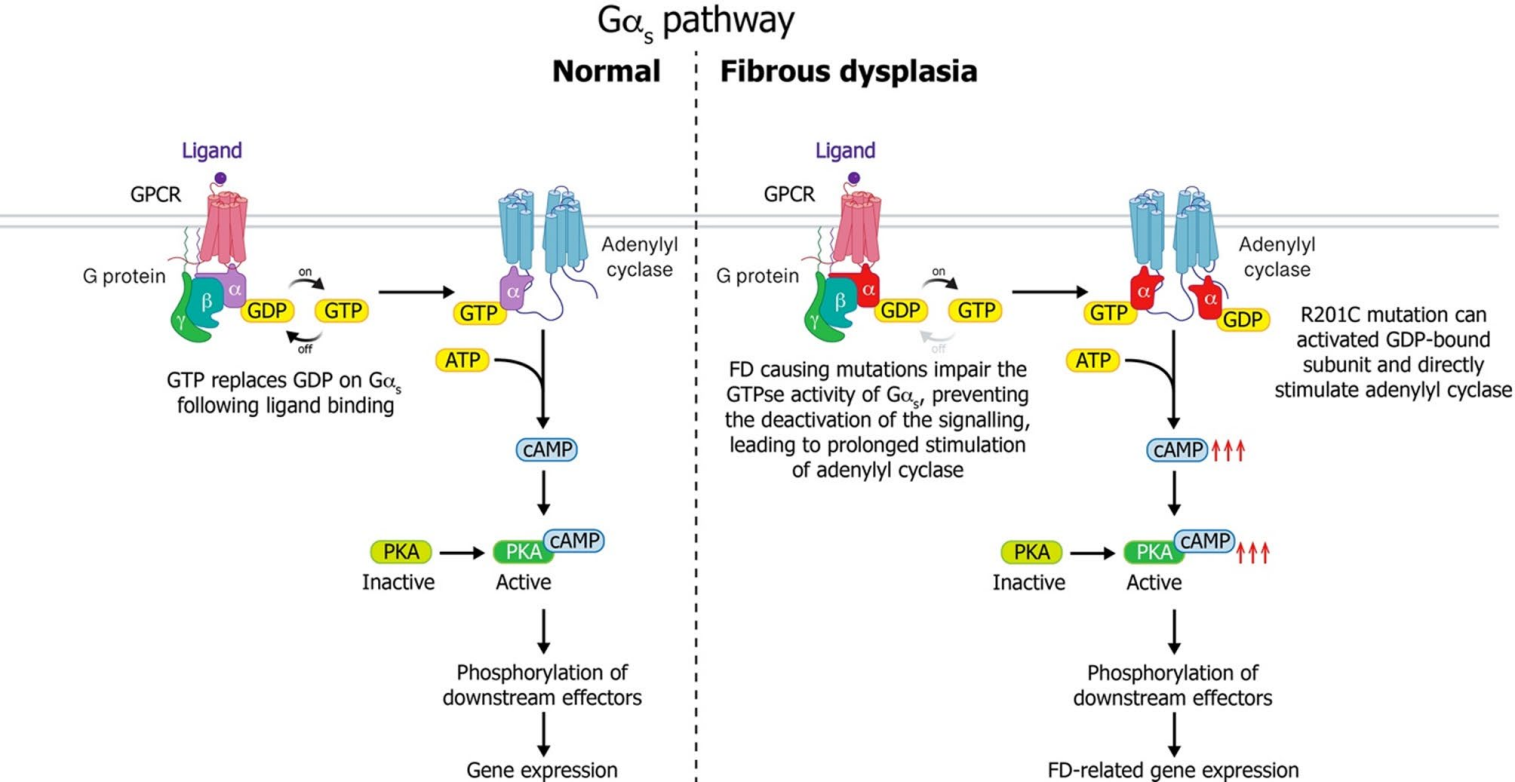
Schematic representation of the α subunit of the stimulatory G protein (Gα_s_) pathway at physiological and FD-related pathological conditions. In the basal state, G_s_ is a heterotrimer composed of GDP-bound Gα_s_ and a βγ heterodimer. Normally, the G protein is activated upon ligand binding to the G protein coupled receptor (GPCR), which leads to replacement of GDP with GTP and dissociation of GTP-bound Gα_s_ from Gβ and Gγ. GTP-bound Gα_s_ activates adenylyl cyclase (AC), leading to intracellular cAMP production. The intrinsic GTPase activity allows the hydrolysis of GTP to GDP and turns off the system. Mutations at R201 and Q227, two residues that are critical for the GTPase activity, lead to constitutive G_s_ activation by impeding the system to turn-off, leading to excess AC activation and cAMP production even in the absence of ligand binding to GPCR

Fibrous dysplasia (FD) of the bone (OMIM #174800, ORPHA: 249) is a rare genetic bone disorder caused by postzygotic activating variants (primarily at the R201 [1] and more rarely at Q227 codons [2]) in *GNAS*, which encodes the α subunit of stimulatory G protein (Gα_s_) [3–5]. These Gα_s_ variants result in impaired GTPase activity, keeping Gα_s_ constitutively active and producing excess cAMP [6] through its prolonged interaction with adenylyl cyclase (AC, Fig. 1). In addition, the R201C variant can bypass the need for GTP binding by directly activating GDP-bound Gα_s_^R201C^ with subsequent direct activation of AC [7] (Fig. 1). This leads to the abnormal bone formation and remodeling that characterizes FD. When the FD bone is accompanied by skin pigmentation and endocrine issues, the combined signs and symptoms are called McCune-Albright syndrome (MAS, OMIM #174800, ORPHA: 562) [8, 9]. As FD is not inherited, the *GNAS* variants are thought to occur after fertilization, resulting in somatic mosaicism [3, 4, 10–12]. This is believed to be due to embryonic lethality of germline *GNAS* activating variants [12], which was confirmed in a recent mouse model of FD/MAS [13, 14]. Notably, however, germline transmission can occur in mice when the mutated *Gnas* cDNA is randomly integrated in the genome [14].

FD lesions are characterized by the growth of a fibro-osseous tissue that replaces the normal bone/bone marrow organ. This causes deformity, fracture, and pain at affected skeletal sites, reducing the quality of life of patients. There is currently no cure, leaving severely affected individuals with physical disabilities such as impaired mobility and chronic pain.

**Fig. 2:**
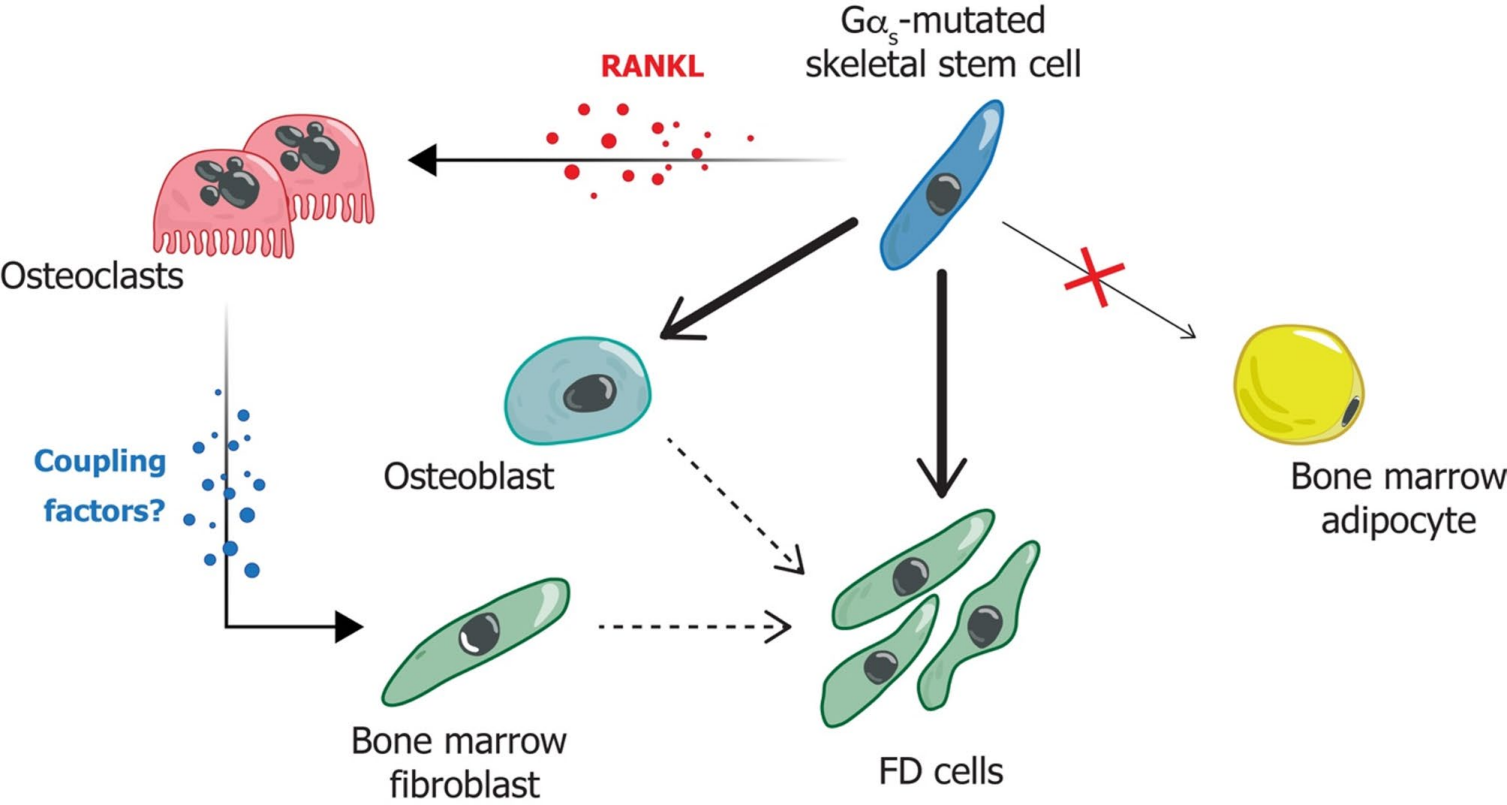
Schematic representation of the hypothetical Gα_s_-mutated SSC function and differentiation capacity in FD. SSCs are thought to be the source of the osteogenic stroma (FD cells) that accumulate in the bone marrow of FD-affected skeletal segments. Mutated SSCs are able to give rise to osteoblasts but are defective in generating bone marrow adipocytes. The FD cells composing the fibrous tissue may also derive from either differentiated osteoblasts or from the expansion of resident bone marrow fibroblasts (dotted arrows) as result of high bone resorption at bone surfaces

Although extensive research has been performed, a detailed understanding of the pathogenesis of FD remains elusive, and further efforts are required to better define the molecular and cellular mechanisms leading to lesions and certain clinical features thereof, including bone pain. It is well established that FD is a disease in which Gα_s_ mutated skeletal stem cells (SSCs) [15] produce an osteogenic stroma that accumulates in the bone marrow (Fig. 2). However, this theory has recently evolved as many observations suggest that mutated SSCs may also induce the expansion of local non-mutated cells [13, 16]. Therefore, a clear understanding of the cell-autonomous and non-cell autonomous effects of the Gα_s_ variants and the interplay between mutated cells and the local microenvironment is fundamental for developing specific therapeutic strategies.

The aim of this review is to detail the critical basic and translational findings that have emerged since the first description of the disease and highlights key concepts and tools for future research in the field.

### FD histopathological features informing research

Defining the histogenesis and histopathology of a disease is fundamental to understanding its clinical expression and natural history. In a research context, the structural changes of affected tissues may provide hints on candidate pathogenetic mechanisms and, in some cases, potential therapeutic targets.

**Fig. 3:**
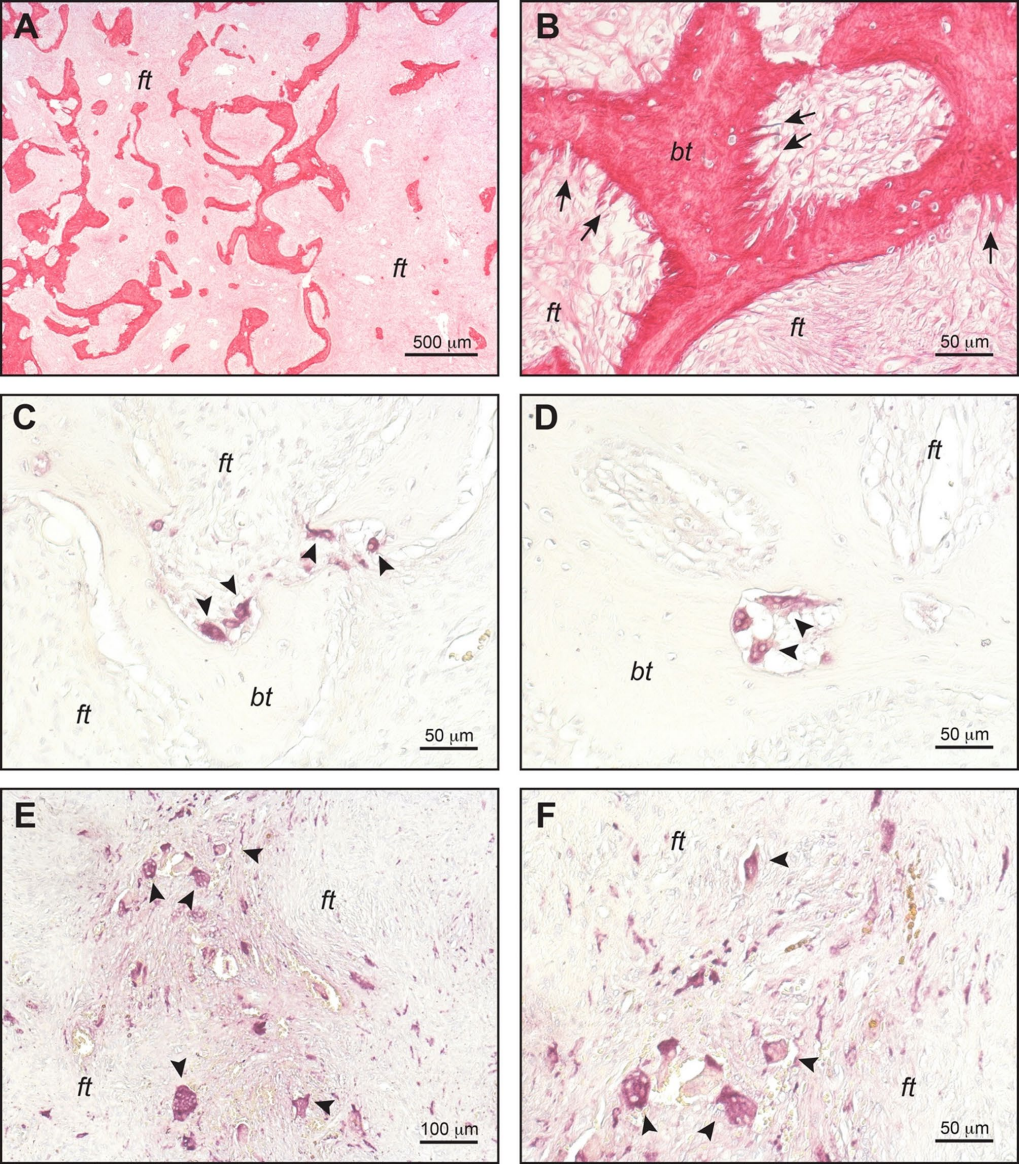
Histology of FD lesions. (**A**) Image of a sirius red stained section showing overall morphology of FD lesions with haphazardly distributed bone trabeculae within the fibrotic marrow. (**B**) High magnification detail of A, showing Sharpey’s fibers (arrows). **C**, **D**) Tartrate resistant acid phosphatase (TRAP) staining showing numerous osteoclasts (arrowheads) on the surfaces of and within (i.e. tunneling resorption) bone trabeculae. **E**, **F**) TRAP staining showing heterotopic osteoclasts (arrowheads) within the fibrous tissue. *ft*: fibrous tissue; *bt*: bone trabecula

FD is currently clinically described as “a benign, medullary, fibro-osseous neoplasm that can be multifocal and is characterized by distorted, poorly organized, and inadequately mineralized bone and intervening fibrous tissue” [17]. Overall, these histological features suggest the alteration of multiple cellular and molecular pathways that participate in bone and bone marrow homeostasis after birth. Indeed, the fibrous tissue results from the expansion of marrow fibroblasts and osteogenic precursors that form abnormal bone and are unable to generate adipocytes or support hematopoiesis (Fig. 2) [1, 18–21]. The osseous component reflects an osteogenic activity that is intrinsically defective, as shown by the deposition of woven and hypo-mineralized bone, with impaired mechanical function, and the haphazard distribution of trabeculae. The newly formed bone is characterized by histological features including abnormally shaped, branched osteoblasts [20, 21] and arrays of collagen bundles perpendicular rather than parallel to its surface [19, 20] (as commonly observed in Sharpey’s fibers [22]) (Fig. 3). Finally, FD is typically associated with dysregulated osteoclastogenesis, which occurs at both orthotopic (i.e., along the trabecular bone surface) and heterotopic (i.e., within the fibrous tissue) sites, that significantly contributes to lesion development and growth. FD lesions have different appearances and behaviors depending on the anatomical segments in which they develop, suggesting that, in addition to the Gα_s_ variant itself, the embryological origin and local factors such as bone remodeling may play a role in pathogenesis [20, 21]. How these processes bring this about needs to be clarified.

In contrast to the WHO definition [17], FD is not limited to the medullary cavity. Indeed, in affected regions, the cortical bone is “trabecularized” for the enlargement of the vascular spaces that are filled by fibrous tissue continuous with that of the intra-medullary lesion. This suggests that abnormal perivascular remodeling in cortical bone may contribute to initiation and/or expansion of FD lesions [20], as also suggested by a recently developed mouse model [16, 23].

Additional changes can be observed in the FD tissue, including, for example, desmoplastic-like changes, myxoid changes, cemented and psammomatous body formation (in particular in gnathic lesions), and cartilaginous differentiation [24–28]. When extensive, these secondary changes must be taken into account not only for potential histological misdiagnosis in the clinic but also for misinterpretation of results in research studies.

Finally, in both FD mouse models and patients [29–33], tissue changes occur in FD lesions exposed to medical treatments, especially denosumab, a humanized monoclonal antibody neutralizing RANKL, which inhibits osteoclastogenesis and converts the fibrous tissue into hyper-mineralized bone.

### Basic research on human FD samples

Human tissue samples are invaluable for unlocking the mysteries of rare diseases. They provide a window into the intricate workings of disease at a cellular and molecular level, allowing researchers to decipher the genetic and proteomic disruptions that underpin these conditions. As a bridge between the laboratory and the clinic, human tissue samples provide critical data that enhances the translation of preclinical findings into potential clinical applications including diagnostic evaluation. During the past decade, there have been tremendous advances in the methodologies that can be applied to human samples. Here, we briefly summarize novel basic research studies using human FD samples that provide insights into disease mechanisms.

#### Ex vivo cell culture of primary human cells

When FD was first appreciated as a distinct pathologic entity, it was originally proposed to be a disease of “bone-forming mesenchyme” [34]. The “mesenchymal” cells forming the fibrotic tissue were later labeled as bone marrow stromal cells (BMSCs) [35], and eventually identified by various names, but essentially recognized as skeletal stem cells [36, 37].

Some of the earliest foundational work on FD human samples was by *ex vivo* culture of these cells. In this context, there have been primarily three approaches: (1) collection of BMSCs from FD lesions [11, 30, 38, 39], (2) modelling FD by genetically modifying BMSCs derived from healthy donors [40, 41] and (3) generation of induced pluripotent stem cells (iPSCs) derived from FD/MAS patients [42, 43]. A main advantage of cell culture models is the ability to perform functional assays under highly controlled experimental conditions. Limitations to these approaches can include artifacts intrinsic to cell culture, such as absence of other cell types that may impact biological responses, unphysiological culture conditions, and the acquisition of cell culture-related spontaneous mutations that may impact cell physiology.

Historically, BMSCs isolated from patients with FD/MAS have been used to understand the pathological basis of FD formation. This included the seminal findings of intra-lesion mosaicism discovered through clonal analysis of primary FD BMSCs [11], as well as cell autonomous and non-cell autonomous signaling induced by the *GNAS* variants [44], including RANKL production [45, 46] and a potential role for vitamin D in FD osteocyte formation [38]. Clonal analyses of the BMSCs also indicated critical roles for cAMP, both as a secondary messenger and as a potential non-cell autonomous regulator of IL-6 production in non-*GNAS* variant bearing cells [47].

#### Genetic and proteomic analysis of primary human samples

Investigation of FD specimens with minimal manipulation is critical to elucidate the underlying molecular mechanisms and identify therapeutic opportunities. Several studies applying genetic tools to primary bulk human FD specimens have identified a number of genes that appear to form a FD gene expression signature [39, 40, 48–53]. This includes the identification that key prominent pathways, such as growth factors, WNT signaling, ephrin ligands, matrix metalloproteinases, and genes encoding components of the cAMP-dependent protein kinase, are very heterogeneous between individual samples [52]. Recently, protein expression profiling on bulk craniofacial samples highlighted high levels of extracellular matrix protein expression in FD, with the potential to distinguish FD from other similar lesions such as cemento-ossifying fibroma [54]. Although these studies have identified a number of different patterns as possibly FD bone-specific, these analyses on bulk tissues have been difficult to generalize into a single disease-specific profile, likely because of the known heterogeneity and mosaicism of the FD bone lesions.

Since FD lesions are highly heterogeneous, comprising multiple cell types (such as fibroblasts, osteoblasts, endothelial, residual hematopoietic cells, etc.), the development of single cell RNA-sequencing (scRNA-seq) provides a unique opportunity to investigate FD specimens. Recently, Kim et al. reported performing scRNA-seq on cells derived from five craniofacial FD lesions [55]. After tissue dissociation to generate single-cell suspensions, cells were cryopreserved and cultured for 2 weeks, prior to scRNA-seq analysis. Although this may change the spectrum of cells and gene expression profiles, the team found that the bone samples showed heterogeneity in cell composition between individual patient samples. The pooled analysis also showed differences in gene expression patterns compared to healthy volunteers, consistent with increased myofibroblast, osteoclast, fibroblastic cell, and macrophage activity. The team subsequently developed an in vitro organoid system that appears to recapitulate several of the highlighted gene expression changes originally identified by the scRNA-seq [55].

One critical area where human samples have been invaluable is the improvement of FD diagnosis. Recent studies have shown that detailed phenotyping [56] and medical image analysis can be used to identify fibrous tissue patterns that are consistent with FD bone lesions, thus providing a tool to improve diagnostic accuracy [57]. In addition, standard genetic diagnosis of FD/MAS from a peripheral blood specimen has been unreliable due to the mosaic nature of the disease. Recent studies indicate that cell-free DNA can be used to identify genetic variants in patients with FD/MAS [43, 58]. Expression of RANKL was also found in human breast tissue of patients with MAS [45], further highlighting the importance of RANKL in FD pathogenesis and suggesting a mechanistic link to the elevated RANKL production that is seen in FD bone.

Analysis of fibro-osseous lesions resembling FD have suggested that FD-related *GNAS* activating variants can also be found in a subset of osteosarcomas [59] providing a genetic link that may explain the numerous prior case reports of increased osteosarcoma risk in patients with FD. Genetics also plays a larger role in improving the specificity of the diagnosis of FD compared to other similarly-appearing lesions [60–62] or between the different genotypes found in FD [63].

### In vivo models of FD

Many experimental studies on FD, which have led to breakthrough discoveries and enabled mechanistic exploration and therapeutic testing not feasible in human patients, have been conducted using in vivo models. These are essential for uncovering disease mechanisms, understanding progression, and developing potential therapeutic strategies.

#### Non-transgenic in vivo models

The cell processes leading to the establishment of a complete bone/bone marrow organ are faithfully recapitulated in the human heterotopic ossicle. This experimental model, generated by transplanting BMSCs with an osteoconductive carrier into immunocompromised mice [35, 64, 65] was used to investigate the ability of Gα_s_-mutated BMSCs to survive and differentiate into osteoblasts *in vivo* [11, 41]. The first study [11] performed with BMSC clonal populations analyzed by gDNA sequencing, led to the identification of both Gα_s_ mutated and WT clonogenic cells within the same FD lesion. Furthermore, it showed that an FD ossicle with abnormal bone could be reproduced only by co-transplanting the two genotypes since no cells could be recovered in transplants made with mutated clones alone. Based on these results, it was concluded that intra-lesion mosaicism is an obligatory condition for Gα_s_ -mutated BMSC survival and function.

However, in a subsequent study, heterotopic FD ossicles were produced by transplanting BMSCs stably transfected with a lentivirus vector carrying the Gα_s_^R201C^ sequence [41] in the absence of their WT counterpart. This provided the first evidence that the Gα_s_ variant *per se* (i.e. as a transgene integrated outside the *GNAS* locus) does not compromise BMSC viability and differentiation. Indeed, a subsequent study has reported that *Adipoq* + marrow stromal cells, a population of mouse multipotent BMSCs [23], was able to generate high amount of lamellar and normally mineralized bone after *in vivo* transplantation when expressing the Gα_s_^R201C^ variant under the defining *Adipoq* promoter [16]. Interestingly, in transplants of *Adipoq-Cre;Gα*_*s*_^*R201C*^ cells, a FD-like fibrous tissue was also observed [16]. Of note, lineage tracing revealed that this tissue did not derive from the Gα_s_-mutated BMSCs but had a distinct origin from local WT cells [16]. Altogether, these results suggest that intralesional mosaicism in FD may have multiple roles, which include, but are not necessarily limited to, cell survival when the Gα_s_ variant is expressed within the *GNAS* locus. Therefore, further studies are required to elucidate the implications of intralesional mosaicism in FD and the molecular mechanisms supporting the interaction between normal and mutated cells.

#### Transgenic mice: overview and comparison of available models

Animal models are extremely valuable tools for disease research as they allow for the study of human diseases and genetic variants in a controlled, *in vivo* experimental setting that bears the closest physiological similarity to humans. Mice share high genetic homology with humans and have similar biological pathways/processes. Molecular genetic tools have been extensively developed and available in mice, such that genetically engineered mouse models can be readily developed to mimic specific human genetic variants, diseases, or disease-related phenotypes, all of which are required to establish causality between genetic changes and disease outcomes.

Prior to the development of mice carrying the causative GNAS variants, some features of FD pathology could be replicated with close approximation in mice with receptor-dependent abnormal activation of the Gα_s_ signaling pathway in osteoblasts (*Col1-caPPR* [66] and *Col1(2.3)/Rs1* mice [67]) and in *Osx-Cre; Catnb*^*Ex3/+*^ mice characterized by enhanced Wnt/β-catenin activity in osteoprogenitor cells [68]. The variety of available FD mouse models provides a breadth of valuable tools to dissect the pathogenesis of FD, while unique features of particular models may help to address specific questions concerning the pathology and clinical presentation of the disease.

The FD phenotype is reproduced in several newer transgenic mouse strains expressing a gain-of-function variant of Gα_s_ in the osteogenic lineage. In the *EF1α-Gnas*^*R201C*^ and *PGK-Gnas*^*R201C*^ mouse strains, the Gα_s_ variant is expressed in the entire osteogenic lineage at all developmental phases, driven by ubiquitous and constitutive promoters [14]. FD skeletal lesions in these mice progress through defined histopathological stages. In contrast with human FD, these models show germline inheritance of the Gα_s_ variant and are not predicted to have genetic mosaicism [14]. Nonetheless, they reproduce the natural history of the human disease as revealed by the postnatal appearance, focal distribution and metachronous development of skeletal lesions. For this reason, they provide useful models to investigate the non-genetic determinants that contribute to the development of FD. Furthermore, they may help to dissect the role of Gα_s_-mutated non-skeletal tissues in the pathology and clinical expression of the disease.

The next generation of transgenic mouse models for FD feature cell type-selective expression of disease-causing *GNAS* mutants. Accumulated evidence supports the concept that FD is caused by activating *GNAS* variant arising in postzygotic stem cells, giving rise to somatic mosaicism. To mimic the pathogenesis of FD, *GNAS* mutants causing FD were selectively expressed in various stem cells and osteoblast progenitors by using tissue-specific and inducible Cre recombinase and TetR-based transgenic systems [69]. This allows for spatial and temporal control over the expression of mutant *GNAS* and, thus, the onset of FD. Use of specific Cre drivers to express human *GNAS*^R201C^ or mouse *Gnas*^R201H^ in skeletal stem cells (*Prrx1-Cre* [13, 70, 71]), osteochondroprogenitors (*Sox9-CreER* [13]) or osteoblast progenitor cells (*Osx-Cre* [13, 71]) successfully replicated the human FD phenotype in limbs and craniofacial bones. In contrast, expression of *Gnas*^*R201C*^ in mature osteoblasts (*Col1a1-Cre* [72]) or *Adipoq*-lineage marrow progenitors (*Adipoq-Cre* [23]*)* either failed to induce FD lesions or induced tail-restricted lesions respectively, demonstrating [73, 74] cell-type specificity for the Gα_s_ variant in FD pathogenesis.

Available FD mouse models have been previously reviewed and are listed in Table 1.

**Table 1:** List of GNAS-based mouse models with reported FD-like phenotypes

Model	Sequence origin	Promoter features	Lineage restriction	FD skeletal phenotype	Time of onset	Other MAS features	REF
EF1α-*Gnas*^*R210C*^	rat	Constitutive	Ubiquitous	All skeletal segments, mostly vertebral column, tail, femurs, humerus, craniofacial bones.	Postnatal, by 6 weeks of age	Myxoma, adenoma, Leydig cell hyperplasia (rare, unpublished observations)	[14]
PGK-*Gnas*^*R210C*^	rat	Constitutive	Ubiquitous	All skeletal segments, mostly vertebral column, tail, femurs, humerus, craniofacial bones.	Postnatal, by 6 weeks of age	Not examined	[14]
*Tet–GNAS* ^*R201C*^ */Prrx1-Cre/LSL-rtTA-IRES-GFP*	human	Tetracyline inducible promoter; lineage traced	mesenchymal progenitors; inducible	Hind and forelimbs, calvaria	Prenatal or postnatal by 7 weeks of age upon Doxycycline induction	Myxoma (rare, unpublished observations)	[70]
*Prrx1-Cre;* *Gnas* ^*f(R201H)*^	mouse	Endogenous locus	mesenchymal progenitors; constitutive	Long bones, calvaria	Prenatal	Not examined	[13]
*Osx-Cre;* *Gnas* ^*f(R201H)*^	mouse	Endogenous locus	Osteoprogenitors; constitutive	Long bones, calvaria	Prenatal	Not examined	[13]
*Sox9-CreER;* *Gnas* ^*f(R201H)*^	mouse	Endogenous locus	Osteochondro progenitors; inducible; Mosaic lesions	Long bones, growth plate adjacent	Postnatal, by p21	Not examined	[13]
*Adipoq-Cre;* *R26-LSL-Gnas* ^*R201C*^	rat	Constitutive	Skeletal stem progenitor cell subset	caudal vertebrae	Postnatal, by 8 weeks of age	Not examined	[16]

#### Transgenic mice: cellular and molecular studies

The different transgenic and knock-in mouse models based on the primary R201 variant that have been developed are invaluable tools for identifying critical cellular and molecular mechanisms underlying various FD phenotypes. Enhanced bone resorption is a prominent histological feature of FD and a major cause of the fragility of affected bones. RANKL expression is increased in FD lesions and some previous studies have shown that RANKL inhibition, achieved either by RANKL neutralizing antibody [29, 30] or small-molecule RANKL inhibitor [75], is effective in modifying the pathology and natural history of the disease, offering potential benefits to patients, especially those with early onset of FD. Using the *EF1α-Gnas*^*R201C*^ mice, it is shown that treatment with an anti-mouse RANKL monoclonal antibodyinduced marked radiographic and microscopic changes at affected skeletal sites in both young and old mice [29]. The anti-RANKL monoclonal antibody treatment induced the deposition of new, highly mineralized bone within the FD lesions that showed a higher mechanical resistance compared to the untreated transgenic mice. The treatment also arrested the growth of established FD lesions and, in young mice, prevented the appearance of new ones. However, after anti-RANKL monoclonal antibody withdrawal, there was phenotypic rebound, and the newly formed bone was remodelled into FD tissue and the disease progression resumed in young mice.

Importantly, Khan et al., and Xu et al., further investigated the cellular and molecular mechanisms underlying FD in the FD conditional KI models. With the *Prrx1-Cre; Gnas*^*f(R201H)/+*^ and *Osx-Cre; Gnas*^*f(R201H)/+*^ mouse models, Xu et al. show that *Gnas*^*R201H*^ expression results in craniofacial phenotypes of FD [71]. Khan et al. [13] and Xu et al. [71] confirmed previous findings in human FD bone marrow stromal cells that the WNT/β-catenin signaling is upregulated and sustained at a higher level by expression of *Gnas* FD variants in vitro and in vivo [13, 68, 71]. It is also demonstrated that Wnt/β-catenin signaling upregulation is at least partially responsible for the FD phenotypes caused by Gα_s_ signaling activation [13, 68, 71]. Genetic inhibition of Wnt/β-catenin signaling or small molecule inhibitors targeting Wnt signaling can effectively alleviate the long bone and cranial phenotypes in mouse FD models caused by *Gnas*^*R201H*^ expression [13, 68, 71].

In addition, while using the FD mouse models to perform cellular and molecular studies, a better understanding of normal and pathological bone development was also gained. It was found that Gα_s_ signaling governs intramembranous ossification by modulating both Wnt/β-catenin and Hedgehog (Hh) signaling pathways [13, 71]. While Gα_s_ signaling activation in FD inhibited osteoblast maturation by activating Wnt/β-catenin signaling and inhibiting Hh signaling, loss of Gα_s_ signaling is found to cause ectopic activation of Hh signaling and YAP transcriptional co-activator, which is an underlying mechanism of progressive osseous heteroplasia (POH, a genetic form of heterotopic ossification caused by loss of function variants in *GNAS*) [76, 77].

In summary, the FD mouse models are invaluable for advancing our mechanistic understanding of the disease and evaluating potential therapies before human clinical trials due to their genetic and physiological similarities to humans. The various mouse models can be used to further dissect the cell-specific effects of Gα_s_ activation in causing FD. They also allow high-throughput experimental studies not feasible in people. These disease mouse models are also invaluable genetic tools to identify the cellular and molecular mechanism underlying normal bone development, which may provide critical insights into other diseases, such as craniofacial osteosarcoma [59, 60].

### Pain in FD/MAS

Pain is a critical aspect of FD due to its potential role as an early indicator of disease activity and its profound impact on the quality of life for FD/MAS patients [78–80]. Craniofacial FD lesions are potential sources of headaches, migraines, and/or orofacial pain [81–83], while deformation of the axial-appendicular skeleton may lead to musculoskeletal pain [84]. Patients frequently report moderate to severe pain, with some cases showing patterns suggestive of a possible neuropathic-like pain phenotype [85, 86]. Anxiety, depression, and decreased quality of life have been linked to pain severity, while neurobiological alterations may perpetuate pain and psychiatric symptoms. There appears to be no consistent correlation between pain severity and disease burden, as assessed by patient reports, bone turnover markers, and radiotracer uptake on 18 F-NaF PET/CT [79, 80, 86–88]. Consequently, surgical or pharmacological approaches that directly target the morphological properties or structural matrix of FD lesions may not consistently result in pain alleviation.

The limited analgesic options for FD/MAS underscore the need for a comprehensive evaluation of current treatments. The efficacy of nonsteroidal anti-inflammatory drugs, acetaminophen, and prescription opioids has historically been unexplored in this population^76,83,84^. Intravenous bisphosphonates are commonly used for patients with unclear pain etiology with variable success. Some FD/MAS patients have reported substantial pain relief with intravenous pamidronate; however, this evidence is derived from observational studies, and no randomized controlled trials have confirmed its efficacy [89]. Notably, a double-blind placebo-controlled study of oral alendronate in FD/MAS patients showed no pain-alleviating effects [90]. In retrospective analyses involving polyostotic FD and MAS cohorts, the clinical benefit (i.e., bone turnover and alleviation of pain) and safety profile of long-term bisphosphonate therapy were described [91]. Yet, the authors noted that determining the analgesic efficacy of bisphosphonates in FD requires further investigation. A recent phase 2 study (NCT03571191) of denosumab showed reduction of lesion activity and improvement of bone formation, though bone-turnover rebound and hypercalcemia remains a concern, particularly in patients with high disease burden [31]. Despite these ongoing studies, chronic pain remains prevalent in FD/MAS patients, highlighting the need to clarify its mechanisms and to identify potential targets for future therapeutic intervention.

Basic research on pain in FD has been sparse. Despite the development of different animal models of FD [73], it is only recently that a potential pain phenotype of these animal models has been explored. Liu et al. [75] were the first to describe behavioral deficits in the FD model developed by Zhao et al. [70]. Mice that developed FD walked and reared significantly less than control mice without FD and FD mice treated with a RANKL inhibitor. Analgesics were not used to determine whether these deficits were pain-related; however, pain may have contributed to the differences observed. Recently, Hopkins et al. [92] have identified nociceptive behaviors in the same model, using analgesics to determine whether nociception is responsible for the behavioral changes and proposing possible nociceptive mechanisms. A range of behavioral tests were employed, such as the grid hanging test, to evaluate muscle strength, and the burrowing test, to assess the ability to empty a tube filled with sand or food pellets and commonly used to assess mouse pain-like behavior. Significant behavioral deficits were observed in both the female and male FD mice compared to the control mice. Impaired grid hanging of the FD mice was improved by morphine, and ibuprofen treatment prior to burrowing improved the performance of the FD mice in this test. This showed that pain-like behavior occurs in a mouse model of FD as lesions develop. Moreover, analyses of tissue samples and the cell secretome of bone marrow stromal cells isolated from the mice suggested several nociceptive mechanisms related to FD pain. Nociceptive nerve fibers, sympathetic nerve fibers, blood vessels, and osteoclasts were present in the FD lesions, and the dorsal root ganglia from male FD mice showed increased staining of activating transcription factor-3 and tyrosine hydroxylase, which are commonly associated with nerve damage in mouse models of chronic pain [93, 94]. Finally, the cell secretome analysis demonstrated a significant increase in inflammatory cytokines, chemokines, and nerve growth factor from the mouse FD cells as compared to the normal bone marrow stromal cells [92]. Thus, several nociceptive mechanisms involving the local FD bone microenvironment, inflammation, and nerve damage were suggested. Accordingly, the expression of neurotrophins such as *BDNF* or *NGF* has been reported in mouse and human FD tissues [32, 52, 95]. However, further studies targeting individual factors (e.g. antibodies targeting a specific cytokine) are required to assess the role of each factor in FD nociception. Pain-like behavior and altered nociceptive responses have also been reported recently in another mouse model of FD, the *EF1α-Gnas*^*R201C*^ [95]. In this model, in which FD lesions spontaneously develop over time generating different patterns of skeletal involvement [14, 95] no correlation between pain, as assessed through several behavioral tests, and disease burden was found [95] supporting evidence reported in human patients [79]. Moreover, no differences have been observed in the pattern of skeletal innervation in periosteum and bone marrow between WT and FD mice. Interestingly, sensory nerve fibers could be detected within mouse FD lesions, but no nerve sprouting or neuroma formation was observed. The absence of Gα_s_- or lesion-dependent nerve sprouting has been confirmed in human FD lesions, thus indicating that pain sensation in FD occurs in the absence of morphological changes of peripheral innervation [95].

### Basic/translational insights informing therapeutics

#### Medical treatments

A transformative discovery in FD/MAS was the recognition of the causative Gα_s_ variants, implying that any tissue that expresses Gα_s_-dependent G-protein coupled receptors is subject to ligand-independent activation of AC/cAMP/PKA pathway (Fig. 1). This discovery provided a unifying molecular explanation as to how melanocyte, gonadal, skeletal, thyroid, pituitary, adrenal, and other pathologies arose and could be seen in a single patient. The timing and location of the acquisition of the postzygotic variant therefore explained the mosaic pattern and severity.

Treatment, then, needed to be directed downstream of Gα_s_ pathway. In the gonads, estrogen and testosterone production could be decreased by inhibiting aromatase, the key enzyme in the steroidogenic pathway [96–98], or by blocking the estrogen receptor with modifiers such as tamoxifen and fulvestrant [99, 100]. Adaptation of treatments proven effective in other hyperactive endocrinopathies, e.g. thionamides for hyperthyroidism [101, 102], adrenal corticosteroid inhibitors, etc. have proven effective in FD/MAS.

While FD is indeed a disease of SSCs, it was also recognized that multinucleated, osteoclast-like cells were prominently observed in many FD samples. These were often seen not associated with bone resorption, but in Ki67-expressing, RANK/RANKL-rich areas of proliferation, especially in younger patients with forming and expanding lesions [46]. These findings led to a very promising clinical trial of the anti-RANKL drug denosumab in adults [30, 31] and an ongoing trial in children (NCT05419050). This study hopes to be the first treatment to prevent the appearance and progression of FD lesions.

Hypophosphatemia has long been recognized as an associated feature of FD. It was initially thought to be the result of Gα_s_ variants in the renal proximal tubule cells. However, the correlation of the degree of renal phosphate loss with skeletal disease burden and the lack of an elevation of cAMP in the urine [103] pointed to a circulating factor produced by FD tissue. The confirmation that FD cells were the source of elevated levels of the phosphaturic hormone FGF23 leading to hypophosphatemia, also led to the discovery that normal bone cells are the physiologic source of FGF23 [104]. This finding has had significant clinical implications and led directly to an ongoing clinical study of the anti-FGF23 monoclonal antibody drug in FD (NCT05509595).

Finally, the multiple mouse models of FD [73, 74] have supported observational hypotheses, helped to unravel the pathophysiology of FD, and led to multiple novel molecular observations, including_:_ preclinical support for RANKL blockade as a treatment for FD [29], a pathogenic role for brain-derived neurotrophic factor in FD [32], different age- lesion- and histology-related molecular profiles of FD lesions [52], and a potential role of Wnt/β-catenin signaling in FD [13]. These remain to be tested in the clinic but are promising and deserve further study.

#### Small molecule inhibitors to lower cAMP

As already mentioned above, the variants in *GNAS* that are responsible for FD/MAS result in the continuous stimulation of transmembrane ACs, leading to increased cAMP production [105, 106] (Fig. 1). Therefore, drugs that lower cAMP production either by blocking Gα_s_-mediated AC activation or by reducing AC activity could mitigate the impact of the mutationally-activated Gα_s_ protein. In one study, Dai and colleagues screened a library of macrocyclic peptides for ones that specifically bind Gα_s_ in either its active or inactive conformation, identifying one molecule for each conformation that is also specific for Gα_s_ relative to other Gα proteins [107]. However, a separate neural-network based computational screening approach [43] failed to identify strong molecular candidates that could specifically target Gα_s_. In addition, cell-permeable AC inhibitors have been identified *via* rational drug design or high-throughput screens using either HEK cells or strains of fission yeast *Schizosaccharomyces pombe* that have been engineered to express individual AC target enzymes [108–110].

## Conclusion and future directions

FD was recognized as a distinct clinical entity shortly after the first reports in 1937 [9, 34]. Nonetheless, for a long time it has remained a poorly defined disease thought to be characterized by “metaplastic” bone. The identification of the Gα_s_ variants in 1991 [4] prompted studies that redefined histogenesis, provided insights into the pathogenesis and led to the establishment of mouse models.

Currently, FD is seen as a disease of SSCs, which abnormal differentiation and functions lead to dysregulated bone remodeling and subversion of the normal bone and bone marrow architecture. This has prompted the development of effective therapies that, although not curative, may help in reducing the progression of the disease.

Nonetheless, key issues remain to be addressed. Further investigation is required on the cell-autonomous and non-cell autonomous role of the Gα_s_-variants to clarify the origin of the fibrous tissue and the interplay between mutated and non-mutated cells, which is crucial for the identification of new therapeutic targets. Moreover, the potential role of the local microenvironment in the development and pathology of FD lesions needs to be further investigated. Finally, studies that clearly define the pathogenetic mechanisms of bone pain in FD are needed to target this symptom that severely affects the patients’ quality of life.

The growing commitment of the scientific community reflected in the numerous research groups around the world actively working on FD and the tight collaboration with patients’ organizations, is expected to provide significant advancement of knowledge in these and other still open questions in the next future.

## Data Availability

Not applicable as no new data were generated for this article.

## Abbreviations

AC: Adenylyl cyclase
BDNF: Brain derived neurotrophic factor
BMSCs: Bone marrow stromal cells
cAMP: Cyclic adenosine monophosphate
FD: Fibrous dysplasia
FGF23: Fibroblast growth factor 23
Gα_s_: α subunit of stimulatory G protein
GDP: Guanosine diphosphate
GPCR: G protein coupled receptor
GTP: Guanosine triphosphate
IL-6: Interleukin 6
MAS: McCune Albright syndrome
NGF: Nerve growth factor
PKA: Protein kinase A
RANKL: Receptor activator of nuclear factor kappa-Β ligand
SSCs: Skeletal stem cells
WHO: World Health Organization
WT: Wild-type
